# Diversity of Rickettsiales bacteria in five species of ticks collected from Jinzhai County, Anhui Province, China in 2021–2022

**DOI:** 10.3389/fmicb.2023.1141217

**Published:** 2023-04-28

**Authors:** Xiaojing Jin, Jiasheng Liao, Qingqing Chen, Junfei Ding, Hongwei Chang, Yong Lyu, Liang Yu, Bohai Wen, Yong Sun, Tian Qin

**Affiliations:** ^1^State Key Laboratory for Infectious Disease Prevention and Control, National Institute for Communicable Disease Control and Prevention, Chinese Center for Disease Control and Prevention, Beijing, China; ^2^Jinzhai County Center for Disease Control and Prevention, Jinzhai, Anhui, China; ^3^Anhui Provincial Center for Disease Control and Prevention, Public Health Research Institute of Anhui Province, Hefei, China; ^4^Lu'an Municipal Center for Disease Control and Prevention, Lu'an, Anhui, China; ^5^Pathogen and Biosecurity, Beijing Institute of Microbiology and Epidemiology, Beijing, China

**Keywords:** Rickettsiales, ticks, Jinzhai County, *Rickettsia*, *Ehrlichia*, *Anaplasma*

## Abstract

The order Rickettsiales in the class Alphaproteobacteria comprises vector-borne pathogens of both medical and veterinary importance. Ticks, as a group, are second only to mosquitoes as vectors of pathogens to humans, playing a critical role in the transmission of rickettsiosis. In the present study, 880 ticks collected from Jinzhai County, Lu'an City, Anhui Province, China in 2021–2022 were identified as belonging to five species from three genera. DNA extracted from individual ticks was examined using nested polymerase chain reaction targeting the 16S rRNA gene (rrs), and the gene fragments amplified were sequenced to detect and identify Rickettsiales bacteria in the ticks. For further identification, the rrs-positive tick samples were further amplified by PCR targeting the *gltA* and *groEL* gene and sequenced. As a result, 13 Rickettsiales species belonging to the genera *Rickettsia, Anaplasma*, and *Ehrlichia* were detected, including three tentative species of *Ehrlichia*. Our results reveal the extensive diversity of Rickettsiales bacteria in ticks from Jinzhai County, Anhui Province. There, emerging rickettsial species may be pathogenic and cause under-recognized diseases. Detection of several pathogens in ticks that are closely related to human diseases may indicate a potential risk of infection in humans. Therefore, additional studies to assess the potential public health risks of the Rickettsiales pathogens identified in the present study are warranted.

## 1. Introduction

Rickettsiales are a group of obligate intracellular gram-negative bacteria in the class Alphaproteobacteria, and many of them have been identified as vector-borne pathogens of medical and veterinary importance. The order Rickettsiales comprises three families: Anaplasmataceae, Rickettsiaceae, and Holosporaceae (Salje, 2021). Humans are exposed to and susceptible to many tick-borne pathogens in the order Rickettsiales, including *Anaplasma phagocytophilum*, which causes human granulocytic anaplasmosis; *Ehrlichia canis, E. chaffeensis*, and *E. ewingii*, which cause different types of ehrlichiosis; *Rickettsia rickettsii*, which causes Rocky Mountain spotted fever (RMSF), and other recently recognized spotted fever group rickettsiae (Nicholson et al., 2010). Recently, globalization, climate warming, and geographical expansion have led to an increase in the incidence and resurgence of human diseases caused by these rickettsial pathogens (Piotrowski and Rymaszewska, 2020; Matos et al., 2022). Rickettsial diseases constitute an emerging public health threat and pose a significant health burden worldwide. However, in past decades, owing to advances in molecular analysis and characterization, an increasing number of novel species/genotypes have been identified, and certain Rickettsiales species previously considered that non-pathogenic have now been linked with human diseases, which facilitates the re-evaluation of the effects of these pathogens on human and animal health (Rar and Golovljova, 2011; Luce-Fedrow et al., 2015; Kim et al., 2022).

Ticks—obligate blood-sucking ectoparasites of vertebrates—are known to carry and transmit various pathogenic microorganisms, including bacterial pathogens in the order Rickettsiales (De La Fuente et al., 2008; Dantas-Torres et al., 2012; Yu et al., 2015). Previous studies suggest that years may elapse between the identification of a potential pathogenic agent in ticks and the detection of associated human infections. This is because the pathogen load and DNA copy number in ticks are much higher than those in human blood (Parola et al., 2013). For example, *Rickettsia sibirica* subsp. *sibirica* BJ-90 was first identified in Chinese ticks (Yu et al., 1993), but the associated human infection was not identified until 22 years later (Jia et al., 2013). Similarly, *Anaplasma phagocytophilum* was first identified in ticks in China in 1997 (Cao et al., 2000), but human granulocytic anaplasmosis was not identified until 10 years later (Zhang et al., 2008). Notably, because of social change and contemporary urbanization, the habitats of humans, animals, and ticks increasingly overlap, which amplifies the risk of human exposure to ticks (Beati et al., 1997; Piotrowski and Rymaszewska, 2020). Therefore, molecular surveillance of pathogens in arthropods is an important aspect of identifying the next rickettsial pathogen that could potentially cause human diseases.

Jinzhai County (Lu'an City) is located on the western border of Anhui Province, at the junction of Anhui, Henan, and Hubei Provinces. This region is ~75% forested and has a humid subtropical monsoon climate, providing suitable habitats for the survival and reproduction of ticks. Previous studies on the molecular detection of tick-borne pathogens in Anhui Province have focused on the detection of *Rickettsia, Hepatozoon, Ehrlichia*, severe fever with thrombocytopenia syndrome virus in goats (Li et al., 2020), and *Anaplasma* spp. in healthy small ruminants (goats and sheep) (Yang et al., 2019). Although there are molecular detection strategies for tick-borne pathogens in local areas, these pathogens have not been extensively and systematically studied. Further investigation and molecular characterization of Rickettsiales bacteria in ticks are necessary. Therefore, the present study aimed to investigate the diversity of tick species and the associated rickettsial pathogens in Jinzhai County over a 2-year monitoring period to provide a scientific basis for the prevention and control of tick-borne rickettsial diseases.

## 2. Materials and methods

### 2.1. Tick collection and identification

During 2021–2022, adult ticks were collected from Jinzhai County, Lu'an city (115.94°E, 31.73°N) in Anhui Province, China. Ticks were collected directly from domestic animals (cattle, dogs, and goats) and grassland. All ticks were first identified based on morphological differences in their capitulum and body by a trained technician using light microscopy (Azmat et al., 2018); the classification was further confirmed by cytochrome c oxidase subunit 1 (*COI*) (Cao et al., 2003) gene sequencing. All ticks were stored at −80°C prior to DNA extraction.

### 2.2. DNA extraction

DNA was extracted from individual adult ticks using a previously described method (Sun et al., 2015). In brief, individual ticks were washed three times with phosphate-buffered saline (PBS) to decontaminate the body surface. The samples were manually homogenized on ice using a 40 mm mortar and pestle (Biosharp, Anhui, China) and then centrifuged for 3 min at 2,500 × *g*. The supernatant was collected for DNA extraction, and genomic DNA was extracted from individual samples using a QIAamp DNA Mini Kit (Qiagen, Hilden, Germany) according to the manufacturer's instructions and eluted in a final volume of 100 μl. All DNA samples were stored at −20°C until experimental use.

### 2.3. Polymerase chain reaction

Preliminary screening of Rickettsiales species was performed using nested or semi-nested PCR. For the detection of *Rickettsia* spp., tick DNA was amplified by nested PCR using primers targeting the 16S ribosomal RNA (16S rRNA) gene (Guo et al., 2016), resulting in the amplification of a 900 bp DNA fragment. To more definitively identify the molecular characteristics of *Rickettsia* pathogens, the positive samples in the preliminary screening were selected for further amplification and sequencing of the citrate synthase (*gltA*) gene, 60 kDa chaperonin (*groEL*) gene, and 16S rRNA (Guo et al., 2016). To identify Anaplasmataceae, molecular screening targeting a fragment of the 16S rRNA gene (Jafar Bekloo et al., 2018) was performed using primers as described previously. To further screen the *Ehrlichia*-positive samples, two sets of PCR primers were used to amplify the sequences of two overlapping fragments of the 16S rRNA gene. In addition, two semi-nested PCRs targeting partial fragments of the *gltA* and *groEL* genes were performed (Lu et al., 2022a), respectively. Subsequently, for *Anaplasma*-positive samples, species-specific primer pairs for five *Anaplasma* species were used for PCR amplification, as previously described (Barlough et al., 1996; Loftis et al., 2006; Rar et al., 2015; Yang et al., 2017, 2018; Guo et al., 2018, 2020). The primers used for amplification are listed in Supplementary Table 1.

The amplification products (5 μl) were verified with 1.0% agarose gel electrophoresis, and the size of the DNA fragments was determined by comparison with standard molecular size DNA ladders. The target fragment amplified by each primer pair was purified using a Gel Extraction Kit (Macherey-Nagel, Duren, Germany). The purified DNA was cloned into pMD19-T plasmid (Takara, Dalian, China). Subsequently, each vector was transformed into *E. coli* cells and its insertion was confirmed by PCR. PCR products with a strong target DNA band were sent for sequencing to Tianyi Huiyuan Biotechnology Company (Beijing, China). DNA fragments shorter than 700 bp were sequenced unidirectionally, whereas fragments longer than 700 bp were sequenced bidirectionally. To avoid cross-contamination, all steps were performed in separate rooms, autoclaved pipettes, and filter tips were used, and the master mix was prepared under a laminar airflow bench (flow rate 0.4 m/s). PCR was performed using Premix Taq Version 2.0 plus dye (Takara, Dalian, China).

### 2.4. Phylogenetic analysis

The obtained DNA sequences were edited and assembled using the SeqMan program (DNASTAR, Madison, WI) and then compared with previously published sequences deposited in GenBank (http://blast.ncbi.nlm.nih.gov/) using the Basic Local Alignment Search Tool (BLAST). The sequences were aligned and analyzed using the multiple sequence alignment program ClustalW (default parameters) as implemented in MEGA, version 11.0. Phylogenetic trees were constructed using the maximum likelihood method implemented in PhyML version 3 (Guindon et al., 2010). The robustness of the phylogenetic trees was assessed based on bootstrap support values obtained from 1,000 replicates; values more than 70% were considered to indicate significant differences. All phylogenic trees were midpoint rooted for clarity only.

## 3. Results

### 3.1. Collection of ticks and detection of Rickettsiales DNA

**Figure 1:**
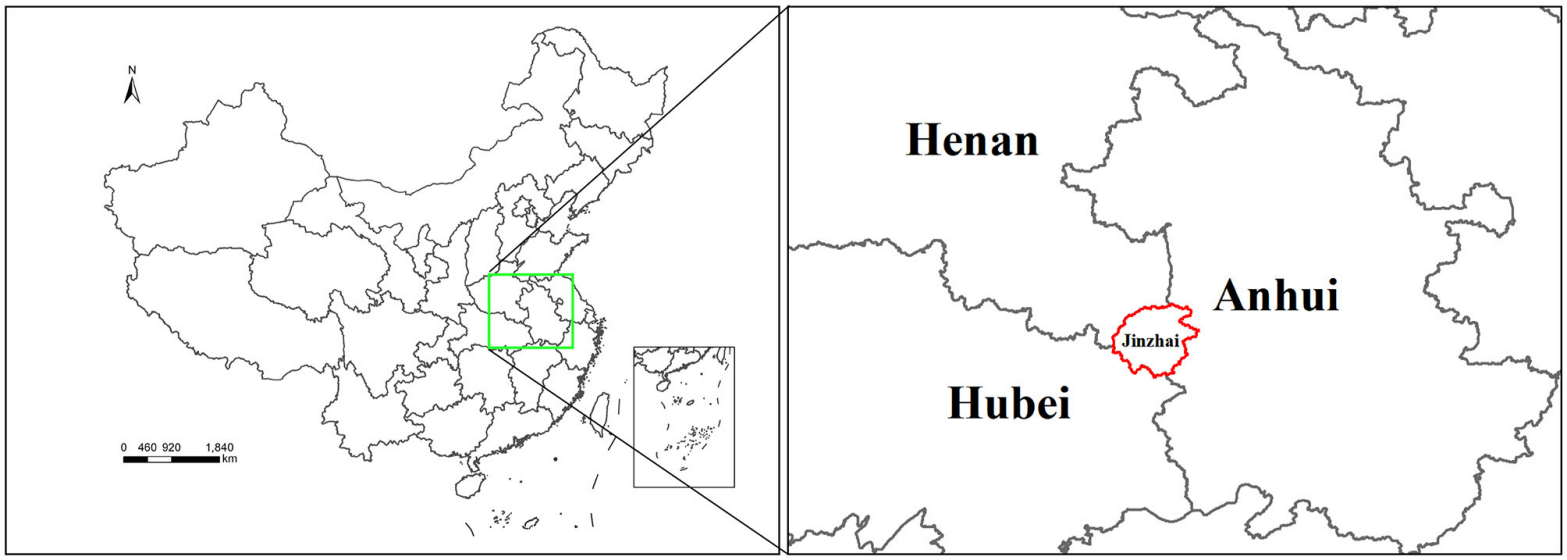
Map showing the location of tick sample collection sites in Jinzhai County, Lu'an City, Anhui Province, China. The region highlighted with a red border indicates the sampling region in this study.

**Figure 2:**
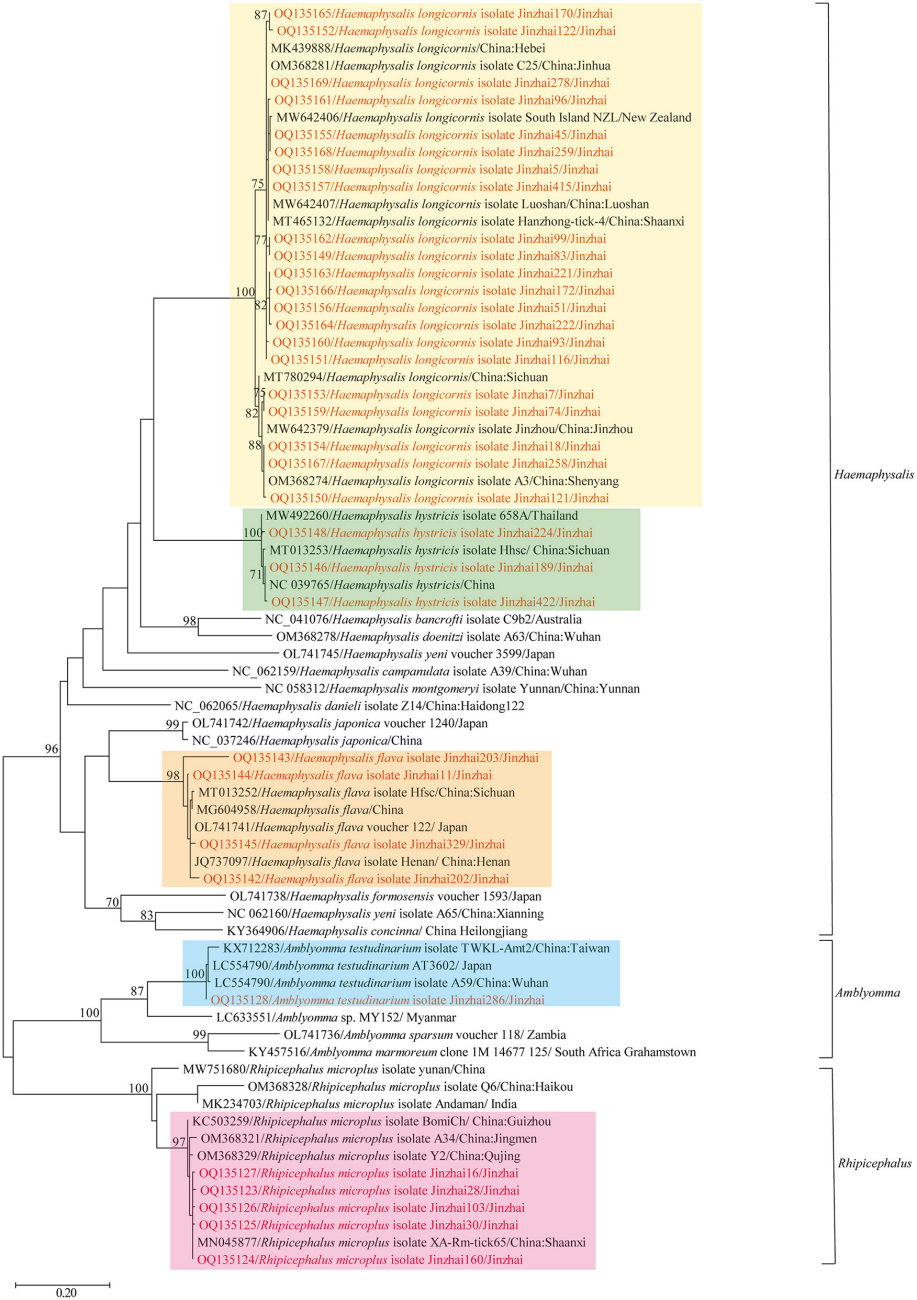
Phylogenetic tree showing the relationship of *Haemaphysalis longicornis, Rhipicephalus microplus, Haemaphysalis hystricis, Haemaphysalis flava*, and *Amblyomma testudinarium* with other tick species. Evolutionary analyses were conducted using PhyML 3.0. The *COI* gene sequences amplified in this study were aligned with other tick *COI* sequences available on GenBank. Sequences obtained in this study are marked in red.

In 2020–2021, 880 ticks were collected from Jinzhai County, Lu'an City, Anhui Province in China (Figure 1). Based on the morphological examination and analysis of *COI* sequences, five tick species belonging to three different genera were identified: *Haemaphysalis longicornis* (62.39%, 549/880), *Rhipicephalus microplus* (27.95%, 246/880), *Haemaphysalis hystricis* (6.25%, 55/880), *Haemaphysalis flava* (2.95%, 26/880), and *Amblyomma testudinarium* (0.45%, 4/880). The phylogenetic tree constructed based on *COI* gene sequences is shown in Figure 2; the *COI* sequences generated in this study were clustered with their respective homologs in five main groups corresponding to five species. The representative *COI* gene sequences from each group were submitted to GenBank (OQ135123–OQ135128, OQ135142–OQ135169). Table 1 presents the GenBank numbers of the Rickettsiales sequences obtained in this study. The species, numbers, and origins of ticks are shown in Supplementary Table 2. The sequences of three different genes were used to construct supertrees, not only to visualize the evolutionary relationships between species but also to better understand the diversity of species (Supplementary Figures 1–3).

**Table 1:** GenBank numbers of Rickettsiales sequences obtained in this study.

**Isolate**	**16S rRNA gene**	***gltA***	***groEL***
*Rickettsia japonica* JZT40	OQ132525	OQ185204	OQ185205
*Rickettsia japonica* JZT135	OQ132526	OQ185240	OQ185206
*Anaplasma platys* JZT2	OQ132527	OQ185241	OQ185207
*Anaplasma bovis* JZT20	OQ132528	OQ185242	OQ185208
*Anaplasma bovis* JZT99	OQ132529	OQ185243	OQ185209
*Anaplasma bovis* JZT240	OQ132530	OQ185265	OQ185210
*Anaplasma bovis* JZT338	OQ132531	OQ185266	OQ185211
*Anaplasma bovis* JZT111	OQ132532	OQ185267	OQ185212
*Anaplasma bovis* JZT161	OQ132533	OQ185268	OQ185213
*Anaplasma bovis* JZT237	OQ132534	-	OQ185214
*Candidatus* Anaplasma boleense JZT106	OQ135107	OQ185244	OQ185215
*Candidatus* Anaplasma boleense JZT21	OQ135108	OQ185245	OQ185216
*Anaplasma capra* JZT12	OQ135109	OQ185246	OQ185217
*Anaplasma capra* JZT94	OQ135110	OQ185247	OQ185218
*Anaplasma marginale* JZT101	OQ135111	OQ185248	OQ185219
*Anaplasma marginale* JZT112	OQ135112	OQ185249	OQ185220
*Anaplasma marginale* JZT4	OQ135113	OQ185250	OQ185221
*Anaplasma marginale* JZT343	OQ135114	OQ185251	OQ185222
*Anaplasma marginale* JZT341	OQ135115	OQ185252	OQ185223
*Anaplasma marginale* JZT53	OQ135116	OQ185253	OQ185224
*Ehrlichia* sp. JZT22	OQ136669	OQ185263	OQ185234
*Ehrlichia* sp. JZT43	OQ136670	OQ185264	OQ185235
*Ehrlichia* sp. JZT19	OQ136671	OQ185270	OQ185236
*Ehrlichia* sp. JZT87	OQ136672	OQ185271	OQ185237
*Ehrlichia* sp. JZT90	OQ136673	OQ185272	OQ185238
*Rickettsia* sp. JZT14	OQ136674	OQ185269	OQ185239
*Ehrlichia* sp. JZT77	OQ136676	OQ185254	OQ185225
*Ehrlichia* sp. JZT88	OQ136677	OQ185255	OQ185226
*Ehrlichia* sp. JZT250	OQ136678	OQ185256	OQ185226
*Ehrlichia* sp. JZT305	OQ136679	OQ185257	OQ185228
*Ehrlichia* sp. JZT234	OQ136680	OQ185258	OQ185229
*Ehrlichia* sp. JZT512	OQ136681	OQ185259	OQ185230
*Ehrlichia* sp. JZT51	OQ136682	OQ185260	OQ185231
*Ehrlichia minasensis* JZT254	OQ136683	OQ185261	OQ185232
*Ehrlichia minasensis* JZT47	OQ136684	OQ185262	OQ185233

### 3.2. *Rickettsia* bacteria detected in ticks

**Figure 3:**
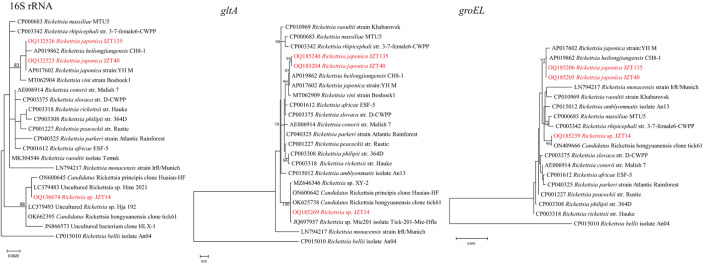
Phylogenetic trees constructed using PhyML 3.0 based on the nucleotide sequences of the 16S rRNA (1213 bp), *gltA* (985 bp), and *groEL* (1051 bp) genes of *Rickettsia* strains. Sequences obtained in this study are marked in red.

Bacteria belonging to the genus *Rickettsia* were detected in *H. longicornis* and *H. flava* (Table 2). Sequencing data and phylogenetic analysis of partial fragments of 16S rRNA, *gltA*, and *groEL* genes showed the presence of *Rickettsia japonica* and *Rickettsia* sp. Specifically, *Rickettsia japonica* was detected in *H. longicornis* and *H. flava*, with positive rates of 1.64% (9/549) and 15.4% (4/26), respectively. The sequences of all *R. japonica* target gene fragments from different tick samples were 100% identical to each other. The 16S rRNA, *gltA*, and *groEL* sequences obtained in this study showed 100%, 99.69%, and 99.81%, respectively, similarity with *R. japonica* strain LA16/2015 (CP047359) and 99.92%, 99.69%, and 99.52%, respectively, similarity with *R. heilongjiangensis* 054 (CP002912), suggesting that the *R. japonica* identified in the present study was closely related to *R. japonica* strain LA16/2015 and *R. heilongjiangensis* 054 (Figure 3).

**Table 2:** Detection of Rickettsiales bacteria in ticks collected from Jinzhai County, China, in 2021–2022.

**Origin**	**Tick species**	***Rickettsia***		***Ehrlichia***				***Anaplasma***				
		***R. japonica***	***Rickettsia*** **sp. JZT14**	***Ehrlichia*** **sp. Yonaguni138**	***E. minasensis***	***E***. **WHBMXZ-43**	***Ehrlichia*** **sp**.	***A. marginale***	***A. bovis***	***A. capra***	***Ca***. **A. boleense**	***A. platys***
Goat	*H. longicornis*	9	0	12	0	0	0	8	39	27	0	0
	*R. microplus*	0	0	0	0	0	0	0	1	1	0	0
	*H. hystricis*	0	0	0	0	0	0	0	3	0	0	0
	*H. flava*	4	0	0	0	0	0	0	0	0	0	0
Cattle	*H. longicornis*	0	0	5	0	0	2	2	0	0	0	0
	*R. microplus*	0	0	0	29	28	9	50	0	0	9	1
	*H. flava*	0	6	0	0	0	0	0	0	0	0	0
Grassland	*H. longicornis*	0	0	2	0	0	0	0	0	0	0	0
	*H. hystricis*	0	0	0	0	0	1	0	2	0	0	0
	*H. flava*	0	2	0	0	0	0	0	0	0	0	0
Total		13	8	19	29	28	12	60	45	28	9	1

Notably, the *Rickettsia* sp. JZT14 obtained from eight *H. flava* collected in 2022 constituted another clade; the sequences of all 16S rRNA, *gltA*, and *groEL* genes of this pathogen from various tick samples were 100% identical to each other. The 16S rRNA gene sequences obtained in this study shared 100% and 99.84% identity with those of uncultured *Rickettsia* sp. Hme_2021 (LC379483) and *Candidatus* Rickettsia hongyuanensis clone tick61 (OK662395), respectively. The *gltA* gene sequences obtained shared 100% identity with those of *Candidatus* Rickettsia principis clone Huaian-HF (ON600642) and 100% identity with those of *Candidatus* Rickettsia hongyuanensis clone tick61 (OK625738). The *groEL* gene sequences obtained from the ticks shared 98.79% identity with those of *Rickettsia peacockii* str. Rustic (CP001227) and 100% identity with *Candidatus* Rickettsia hongyuanensis clone tick61 (ON409666). *Rickettsia* sp. JZT14 identified in the present study was closely related to *Candidatus* Rickettsia hongyuanensis clone tick61 (Lu et al., 2022b) and *Candidatus* Rickettsia principis clone Huaian-HF (Qi et al., 2022).

### 3.3. *Anaplasma* bacteria detected in ticks

In the present study, bacteria belonging to the genus *Anaplasma* were identified in *H. longicornis, R. microplus*, and *H. hystricis* (Table 2). Genetic and phylogenetic analyses revealed that the sequences were clustered into five main clades corresponding to five *Anaplasma* species: *Anaplasma marginale, A. bovis, A. capra, A. platys*, and *Candidatus* A. boleense.

**Figure 4:**
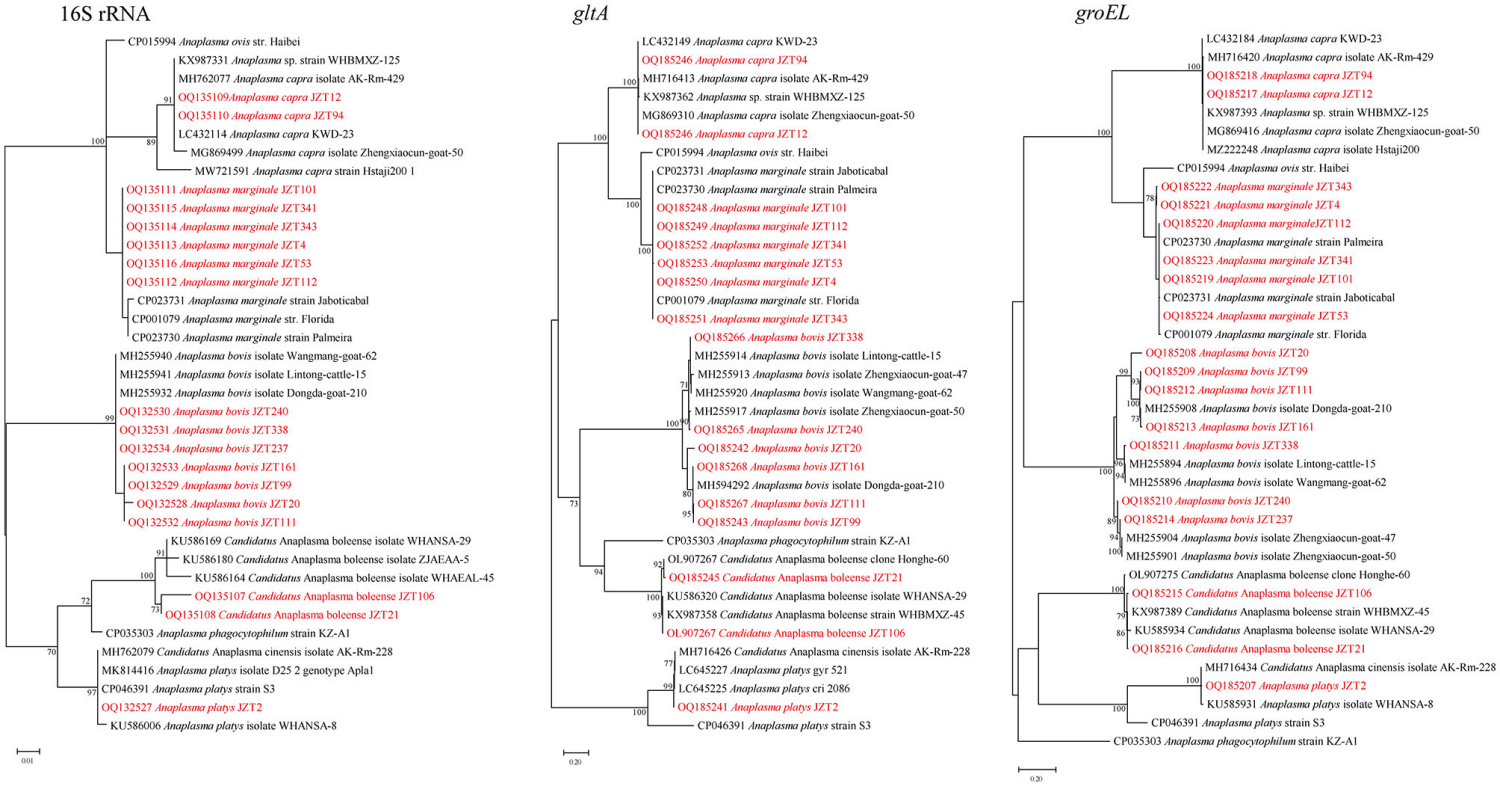
Phylogenetic trees constructed using PhyML 3.0 based on the nucleotide sequences of the 16S rRNA (837–1,234 bp), *gltA* (711–1,102 bp), and *groEL* (822–1,371 bp) genes of *Anaplasma* strains. Sequences obtained in this study are marked in red.

Specifically, *A. marginale* was detected in *H. longicornis and R. microplus*, with positive rates of 1.8% (10/549) and 20.3% (50/246), respectively. In the 16S rRNA phylogenetic tree (Figure 4), all sequences were clustered together with those of *A. marginale* strains obtained from cattle (De Andrade et al., 2004) and dairy bovines from Brazil (https://www.ncbi.nlm.nih.gov/bioproject/348690). The topology of the *gltA* and *groEL* gene sequence-based phylogenetic trees was similar to that of the 16S rRNA sequence-based phylogenetic tree. The 16S rRNA, *gltA*, and *groEL* gene sequences from the strains detected in the present study shared 100%, 99.64–100%, and 99.88–100%, respectively, identity with previously reported *A. marginale* sequences. *Candidatus* A. boleense was detected in *R. microplus*, with a positive rate of 3.7% (9/246). In the 16S rRNA gene sequence phylogenetic tree, the sequences showed a close relationship with the species *Ca*. A. boleense detected in mosquitoes collected in China (Guo et al., 2016). The 16S rRNA, *gltA*, and *groEL* genes from the strains detected in ticks in the present study showed 99.29–99.67%, 98.6–100%, and 99.54–99.77%, respectively, similarity with *Ca*. A. boleense strains reported in China. *Anaplasma capra* was detected in *H. longicornis* and *R. microplus*, with positive rates of 4.9% (27/549) and 1.0% (1/96). The 16S rRNA gene sequences of *A. capra* obtained in this study were 100% identical to those of *A. capra* KWD-23 (LC432114), *A. capra* isolate AK-Rm-429 (MH762077), and *Anaplasma* sp. strain WHBMXZ-125 (KX987331) published in GenBank, indicating the same genetic lineage. In the *groEL* and *gltA* phylogenetic trees, the sequences identified in the present study showed a close relationship with those of *A. capra* strains detected in ticks collected in Wuhan (Lu et al., 2017) and Shanxi (Guo et al., 2019) provinces and in goats in Shaanxi province in China (Guo et al., 2018) and in water deer in Korea (Amer et al., 2019). *Anaplasma bovis* was detected in *H. longicornis, R. microplus*, and *H. hystricis*, with positive rates of 7.1% (39/549), 0.4% (1/246), and 9.1% (5/55), respectively. The 16S rRNA, *gltA*, and *groEL* genes showed 99.55–99.89%, 92.66–99.66%, and 95.83–99.72%, respectively, similarity with the previously reported sequences *A. bovis* strains. *Anaplasma platys* was detected in *R*. *microplus*. The 16S rRNA gene sequence of *A. platys* detected in this study was 100% identical to that of *A. platys* identified in dogs and mosquitoes in Saint Kitts and Nevis and China and that of *Candidatus* A. cinensis detected in ticks in China. The *gltA* and *groEL* phylogenetic trees showed that the bacterial strains detected in ticks collected in this study are clustered in the same group with *A*. *platys* and *Candidatus* A. cinensis. *Anaplasma platys* strain S3 formed a neighbor cluster with *A. platys* JZT2.

### 3.4. *Ehrlichia* bacteria detected in ticks

Bacteria belonging to the genus *Ehrlichia* were detected in *H*. *longicornis, R*. *microplus*, and *H*. *hystricis* (Table 2). The phylogenetic trees of sequences of the target genes amplified by PCR in this study showed that two classes of trees about *Ehrlichia* bacteria were established, corresponding to three known and three tentative species of *Ehrlichia*.

**Figure 5:**
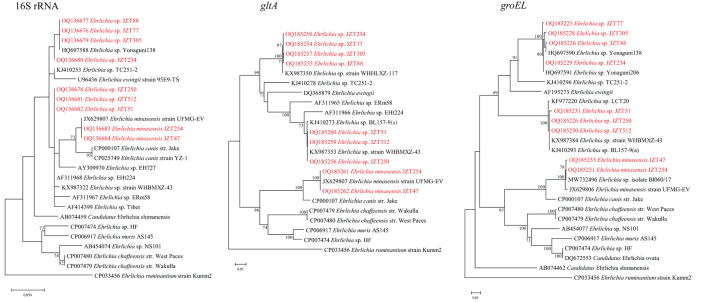
Phylogenetic trees constructed using PhyML 3.0 based on the nucleotide sequences of the 16S rRNA (1,198 bp), *gltA* (977 bp), and *groEL* (1,111 bp) genes of *Ehrlichia* strains. Sequences obtained in this study are marked in red.

*Ehrlichia minasensis* was detected in *R*. *microplus* (11.8%, 29/246). The 16S rRNA gene sequences obtained in this study were 100% similar to that of the *E*. *minasensis* strain UFMG-EV published in GenBank, whereas the *gltA* and *groEL* sequences were both most closely related to the corresponding sequences of *E. minasensis* strain UFMG-EV (99.90% similarity for *gltA*, 99.74–99.91% similarity for *groEL*). In addition to *E. minasensis*, some less well-characterized strains were identified. *Ehrlichia* sp. JZT512, JZT51, and JZT250, which were more closely related to *Ehrlichia* sp. strain WHBMXZ-43, was identified in *R. microplus*, with a positive rate of 11.4% (28/246). The obtained 16S rRNA gene sequences were 100% similar to those of *Ehrlichia* sp. strain WHBMXZ-43, whereas the obtained *gltA* and *groEL* sequences showed a high similarity to the corresponding sequences obtained from this strain (100% similarity for *gltA* and 99.91–100% similarity for *groEL*). *Ehrlichia* sp. JZT77, JZT88, JZT234, and JZT305, which were more closely related to *Ehrlichia* sp. Yonaguni 138/206, were detected in *H. longicornis*, with a positive rate of 3.5% (19/549), respectively. In the 16S rRNA gene phylogenetic tree (Figure 5), the sequences clustered together with those of *Ehrlichia* sp. Yonaguni 138 was identified in *H. longicornis* from Japan (Kawahara et al., 2006). In the *groEL* gene sequence phylogenetic tree, although the sequences from *Ehrlichia* sp. JZT88 and JZT305 clustered together with those of *Ehrlichia* sp. Yonaguni138, the sequences from *Ehrlichia* sp. JZT77 and JZT234 were more closely related to those of *Ehrlichia* sp. Yonaguni 206. In the *gltA* gene sequence phylogenetic tree, the sequences detected in the present study clustered together and showed a relatively close relationship with those of *Ehrlichia* sp. strain WHHLXZ-117 (Lu et al., 2017) identified in ticks collected in China.

**Figure 6:**
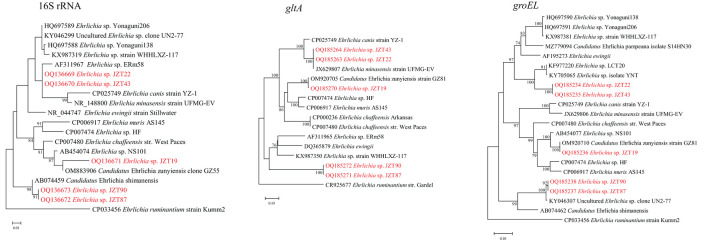
Phylogenetic trees constructed using PhyML 3.0 based on the nucleotide sequences of the 16S rRNA (1,433 bp), *gltA* (978–1,234 bp), and *groEL* (1,224 bp) genes of *Ehrlichia* strains. Sequences obtained in this study are marked in red.

Notably, we found that several sequences detected only in the ticks collected in 2022 differed from those deposited in GenBank. To further ascertain the genetic evolutionary relationship and relationship between the *Ehrlichia* bacteria identified in this study and previously reported *Ehrlichia* bacteria (based on NCBI sequences), phylogenetic analyses were conducted (Figure 6) based on longer fragments of the 16S rRNA, *gltA*, and *groEL* genes. The analysis possibly demonstrated the presence of three potentially novel *Ehrlichia* variants. Two uncultured *Ehrlichia* sp. (JZT87 and JZT90) from *H. longicornis* in all phylogenetic trees were observed to be closely related to *Candidatus* Ehrlichia shimanensis, with 99.78–99.93% and 93.93–93.97% similarity in 16S rRNA and *groEL* sequences, respectively. The phylogenetic trees based on the sequences of 16S rRNA, *gltA*, and *groEL* showed that *Ehrlichia* sp. JZT19 detected in *H. hystricis* was more closely related to species included in clades associated with *E. chaffeensis*, including *Ehrlichia* sp. HF (98.46%, 88.32%, and 94.82% similarity for 16S rRNA, *gltA*, and *groEL*, respectively), *E. muris* AS145 (98.67%, 88.95%, and 94.66% similarity for 16S rRNA, *gltA*, and *groEL*, respectively), and *E. chaffeensis* str. West Paces (98.88%, 86.13%, and 95.06% similarity for 16S rRNA, *gltA*, and *groEL*, respectively). In addition, the phylogenetic trees based on the sequences of 16S rRNA, *gltA*, and *groEL* showed that two uncultured *Ehrlichia* sp. (JZT22 and JZT43 from *R. microplus*) formed a unique clade, suggesting that they are closely related to *Ehrlichia* sp. ERm58, *Ehrlichia minasensis* strain UFMG-EV, and *Ehrlichia ewingii*.

## 4. Discussion

In the present study, the diversity of ticks collected from Jinzhai County and the rickettsial pathogens they harbored were examined using molecular techniques (PCR, DNA sequencing, and phylogenetic analysis) to improve the understanding of the genetic diversity of local ticks and tick-borne Rickettsiales bacteria. A total of 880 ticks collected from Jinzhai County in 2021–2022 were identified as belonging to *H. longicornis, R. microplus, H. hystricis, H. flava, and A. testudinarium*. PCR analyses targeting Rickettsiales genes (16S rRNA, *gltA*, and *groEL*) identified 13 species/variants belonging to the order Rickettsiales and the genera *Rickettsia, Anaplasma*, and *Ehrlichia*, including three tentative species of *Ehrlichia*. Importantly, many of the detected Rickettsiales species were closely related to known human and animal pathogens. Collectively, our findings suggest that different rickettsial species or genotypes are distributed in the region and reveal extensive diversity of Rickettsiales bacteria in ticks collected from this region.

In the present study, two *Rickettsia* spp.—including *R. japonica* and *Rickettsia* sp. JZT14—were detected in the ticks. *R. japonica*, the causative agent of Japanese spotted fever (JSF), has become increasingly prevalent in China in recent years (Li et al., 2018, 2019a; Lu et al., 2018; Yin et al., 2018; Li and Liu, 2021). Moreover, the first human death associated with *R. japonica* infection has recently been reported in Hubei Province in central China (Teng et al., 2023), suggesting that *R. japonica* poses a potential threat to human health, with serious consequences. In this study, phylogenetic analyses of bacterial sequences obtained from the ticks suggested the presence of *R. japonica* in the region, revealing a potential risk of JSF for local populations. In addition, we also identified a variant “*Rickettsia* sp. JZT14.” According to gene sequence-based criteria (Fournier et al., 2003), for a pathogen to be classified as a novel species, it should fulfill five conditions; for example, the sequence similarity of *gltA* with the species that is validated to be the most homologous should not exceed 99.9%. The similarity of both 16S rRNA and *gltA* genes in this study was higher than this criterion. Therefore, we concluded that it was not a novel species. However, additional studies are needed to verify this finding, for example, by obtaining the whole-genome sequence of the organism by isolation and culture as a way to accurately classify the rickettsial pathogen. To date, the efforts by our research group to this end have not succeeded in obtaining isolates. Emerging rickettsial bacteria may be pathogenic and cause under-recognized diseases that threaten the health of local populations. Therefore, further studies are warranted to assess the potential public health risks of this pathogen.

*Anaplasma* species represent globally distributed tick-borne bacteria of veterinary and public health importance. In the present study, five bacterial species belonging to the genus *Anaplasma* were identified: *A. marginale, A. bovis, A. capra, A. platys*, and *Ca*. A. boleense. *Anaplasma marginale* is the causative agent of tick-transmitted rickettsial diseases of cattle (Kocan et al., 2003). Although this pathogen commonly infects cattle, causing bovine anaplasmosis, it has also been detected in non-bovine species such as sheep (Yousefi et al., 2017) and goats (Barbosa et al., 2021). In the present study, phylogenetic analyses showed that the *A. marginale* sequences detected in ticks were clustered together in a single clade, suggesting that these sequences are conserved across isolates from different regions and different hosts.

*Anaplasma capra, A. bovis*, and *A. platys* detected in this study have been identified as human and animal pathogens (Maggi et al., 2013; Arraga-Alvarado et al., 2014; Li et al., 2015a; Lu et al., 2019). *Anaplasma capra* is widely distributed in China, Turkey, Kyrgyzstan, and Korea and has been identified in *H. longicornis*, water buffalo, sheep, goats, and dogs (Lee et al., 2005; Zhang et al., 2017; Han et al., 2019; Shi et al., 2019; Sahin et al., 2022). *A. capra* was first identified in 2015 as a novel *Anaplasma* species found to cause human infection in China (Li et al., 2015a). The present study is the first to detect *A. capra* DNA in ticks collected from goats in Anhui Province, confirming the presence of this pathogen in this region and the potential risk to local residents. In 2017, a case of human anaplasmosis caused by *A. bovis*, which was previously thought to be a bovine-specific pathogen, was reported in China (Lu et al., 2019). In previous studies, *A. bovis* infection has been reported in domestic small ruminants in Anhui Province (Yang et al., 2019), suggesting that *A. bovis* is commonly found in Anhui Province. *Anaplasma platys*, a widely distributed pathogen, is the causative agent of canine cyclic thrombocytopenia affecting cats (Lima et al., 2010) and some ruminants such as cattle, goats, camels, buffalo, and red deer (Chochlakis et al., 2009; Dahmani et al., 2015; Li et al., 2015b; Lorusso et al., 2016a,b; Machado et al., 2016). In 2013, the first case of *A. platys* infection in humans was confirmed by DNA sequencing (Maggi et al., 2013). Another study (Arraga-Alvarado et al., 2014) provided additional molecular evidence for *A. platys* infections in humans, suggesting that *A. platys* is a potential zoonotic pathogen. A previous study in Wuhu reported the presence of *A. platys* in stray dogs (Yang et al., 2020). In the present study, we detected *A. platys* DNA in ticks collected from cattle, indicating a potential risk of zoonotic infections in humans who are in regular contact with their cattle. Three of the five pathogenic bacterial species detected in the present study—*A. marginale, A. bovis*, and *A. capra*—were detected in samples collected in both 2021 and 2022, indicating the sustained presence of *Anaplasma* infection at the study sites. In addition, the transmission intensity of some pathogens belonging to the order Rickettsiales fluctuates throughout the year and from year to year, even in the same region. Long-term surveys with a large sample size are needed to investigate the dynamic changes in pathogen prevalence.

*Ehrlichia* species are responsible for life-threatening emerging human zoonoses and diseases of veterinary concern worldwide (Esemu et al., 2011). The present study reports the detection of well-known pathogens and unclassified genetic variants in this genus. *Ehrlichia minasensis*, a new genotype phylogenetically close to *Ehrlichia canis* (Cruz et al., 2012; Aguiar et al., 2014), has been reported in not only bovines, cervids, and dogs but also several tick species from China, Canada, Brazil, France, Pakistan, Ethiopia, and Israel (Li et al., 2019b). Although the specific species of ticks that transmit this pathogen has not been identified, the detection and transmission of *E. minasensis* by *R. microplus* have been documented (Cabezas-Cruz et al., 2016). The present study is the first to report that *E. minasensis* exists in Anhui Province. The detection of this species suggests that it circulates in cattle in Jinzhai County, and a seroepidemiological investigation of local cattle is needed to understand its prevalence in affected animals. In the present study, uncultured strains belonging to two *Ehrlichia* spp. (*Ehrlichia* sp. JZT77, JZT88, JZT234, and JZT305 and *Ehrlichia* sp. JZT512, JZT51, and JZT250) were detected in this study. As shown in Figure 5, *Ehrlichia* sp. JZT77, JZT88, JZT234, and JZT305 were clustered together with *Ehrlichia* sp. Yonaguni 138/206 found in *H. longicornis* from Japan (Kawahara et al., 2006) and *Ehrlichia* sp. strain WHHLXZ-117 (Lu et al., 2017) found in ticks from China. In contrast, *Ehrlichia* sp. JZT512, JZT51, and JZT250 were more closely related to *Ehrlichia* sp. strain WHBMXZ-43 identified in *R. microplus* from Wuhan, China. Nevertheless, the pathogenicity of these *Ehrlichia* strains in humans has not been reported. As previously mentioned, the present study demonstrated three newly identified *Ehrlichia* members. These tick-borne pathogens may represent a serious public health threat to humans. In addition, many pathogens that were previously considered non-pathogenic are now commonly associated with human diseases (Jia et al., 2013). Thus, even microorganisms of unknown pathogenicity pose a potential and considerable risk of causing emerging diseases. Therefore, isolating new microorganisms from these tick species and examining their potential pathogenicity in humans is of great significance. In the future, our research group aims to isolate the Rickettsiales bacteria identified in the present study to examine their pathogenicity.

It should be noted that our study has several limitations. We cannot conclude whether the entirety of the microbial DNA detected originated from the ticks or whether the results may be influenced by the previous blood meal of the ticks. In addition, the method used for phylogenetic tree construction in this study was a maximum likelihood, which is the optimal algorithm when the evolutionary model is chosen reasonably; however, it is computationally intensive and time-consuming.

## 5. Conclusion

In the present study, we collected tick specimens for two consecutive years to detect tick-borne Rickettsiales pathogens and recognize the diversity of Rickettsiales in the study area. Early detection and identification of potential rickettsial pathogens are essential for improving response measures and preventing associated zoonotic diseases. The present findings demonstrate the need for constant surveillance of Rickettsiales pathogens in ticks in China to assess the potential risk of transmission of disease-causing pathogens or colonizing species in vector organisms to humans.

## Data availability statement

The datasets presented in this study can be found in online repositories. The names of the repository/repositories and accession number(s) can be found below: https://www.ncbi.nlm.nih.gov/genbank/, OQ132525-OQ132534, OQ135107-OQ135116, OQ135123-OQ135128, OQ135142-OQ135169, OQ136669-OQ136674, OQ136676-OQ136684, and OQ185204-OQ185272.

## Author contributions

XJ, JL, TQ, and YS provided the idea, designed the study, and drafted the manuscript. QC, JD, HC, and YL performed the sample collection and laboratory work. LY performed the experimental data analysis. TQ, YS, and BW revised the manuscript. All authors contributed to the article and approved the submitted version.

## Supplementary material

Supplementary Figure 1 Phylogenetic trees based on partial Rickettsiales 16S rRNA sequences using the ML method with PhyML 3.0. Sequences of *Rickettsia* strains obtained in this study are marked in red; sequences of *Ehrlichia* strains obtained in this study are marked in blue; and sequences of *Anaplasma* strains obtained in this study are marked in green.

Supplementary Figure 2 Phylogenetic trees based on partial Rickettsiales *gltA* sequences using the ML method with PhyML 3.0. Sequences of *Rickettsia* strains obtained in this study are marked in red; sequences of *Ehrlichia* strains obtained in this study are marked in blue; and sequences of *Anaplasma* strains obtained in this study are marked in green.

Supplementary Figure 3 Phylogenetic trees based on partial Rickettsiales *groEL* sequences using the ML method with PhyML 3.0. Sequences of *Rickettsia* strains obtained in this study are marked in red; sequences of *Ehrlichia* strains obtained in this study are marked in blue; and sequences of *Anaplasma* strains obtained in this study are marked in green.

Supplementary Table 1 The primers used for the amplification of 16S, *gltA, and groEL* genes by PCR, nested PCR, or semi-nested PCR.

Supplementary Table 2 The species, numbers, and origins of ticks collected from Jinzhai County.
