# Supplementary material for: Diversity of Rickettsiales bacteria in five species of ticks collected from Jinzhai County, Anhui Province, China in 2021–2022
Source: Front Microbiol. 2023 Apr 28;14:1141217. doi: 10.3389/fmicb.2023.1141217 (PMC10175684; doi:10.3389/fmicb.2023.1141217)
Supplement: Supplementary Figure 1 — Phylogenetic trees based on partial Rickettsiales 16S rRNA sequences using the ML method with PhyML 3.0. Sequences of Rickettsia strains obtained in this study are marked in red; sequences of Ehrlichia strains obtained in this study are marked in blue; and sequences of Anaplasma strains obtained in this study are marked in green. [file Data_Sheet_1.docx]

Supplementary Material

**Diversity of Rickettsiales bacteria in five species of ticks collected from Jinzhai County, Anhui Province, China, in 2021–2022**

Xiaojing Jin^1†^, Jiasheng Liao^2†^, Qingqing Chen ^3^, Junfei Ding^2^, Hongwei Chang^4^ ,Yong Lyu ^4^ ,Liang Yu^2^, Bohai Wen^5^*,Yong Sun^3^* and Tian Qin^1^*

^1^State Key Laboratory for Infectious Disease Prevention and Control, National Institute for Communicable Disease Control and Prevention, Chinese Center for Disease Control and Prevention, Beijing, China

^2^Jinzhai County Center for Disease Control and Prevention, Jinzhai, Anhui, China

^3^Anhui Provincial Center for Disease Control and Prevention, Public Health Research Institute of Anhui Province, Hefei, China

^4^Lu'an Municipal Center for Disease Control and Prevention, Lu'an, Anhui, China

^5^Pathogen and Biosecurity, Beijing Institute of Microbiology and Epidemiology, Beijing, China

*** Correspondence:***Tian Qin*
[qintian@icdc.cn](mailto:qintian@icdc.cn)

*Yong Sun*

[biosunyong@163.com](mailto:biosunyong@163.com)

*Bohai Wen*

bohaiwen@sohu.com

# Supplementary Tables

**Supplementary Table 1.** The primers used for amplification of 16S, *gltA*, and *groEL* genes by PCR, nested PCR or semi-nested PCR.

| Bacteria | Cycle | | Gene | Primer Sequence (5’-3’) | Anticipated amplicon length | Reference |
| --- | --- | --- | --- | --- | --- | --- |
| *Rickettsia* | 1,2 | 16S | | GTACGGAATAACTTTTAGAAAT(+) | 900 bp | (Guo et al., 2016) |
|  | 1 | 16S | | CATGATGACTTGACRTCGT(-) |  |  |
|  | 2 | 16S | | CATCTCACGACACGAGCTG(-) |  |  |
|  | 1 | 16S | | GAAGGCGRTCATYTRGGCT(+) | 600 bp |  |
|  | 2 | 16S | | GRTCATYTRGGCTRCAACTG(+) |  |  |
|  | 1 | 16S | | CTGCCTCTTGCGTTAGCT |  |  |
|  | 1 | gltA | | CCGGGYTTTATGTCTACTGC(+) | 1100 bp |  |
|  | 2 | gltA | | CTTTATGTCTACTGCKTCTTG(+) |  |  |
|  | 1,2 | gltA | | AGCTGTCTWGGTCTGCTGATT(-) |  |  |
|  | 1 | groEL | | CCATTACATGATAGAATTGCAAT(+) | 1100 bp |  |
|  | 2 | groEL | | GAATTGCAATAAAGCCTATCG(+) |  |  |
|  | 1, 2 | groEL | | CCATCATTGCTTTTCTTCTATC(-) |  |  |
| Anaplasmataceae | 1 | 16S | | GAACGAACGCTGGCGGCAAGC(+) | 450 bp | (Jafar Bekloo et al., 2018) |
|  | 1 | 16S | | AGTAYCGRACCAGATAGCCGC(-) |  |  |
|  | 2 | 16S | | TGCATAGGAATCTACCTAG(+) |  |  |
|  | 2 | 16S | | CTAGGAATTCCGCTATCCTCT(-) |  |  |
| *A. capra* | 1 | 16S | | TCCTGGCTCAGAACGAACGCTGGCG(+) | 1261 bp | (Barlough et al., 1996) |
|  | 1 | 16S | | AGTCACTGACCCAACCTTAAATGGCTG(-) |  |  |
|  | 2 | 16S | | GCAAGTCGAACGGACCAAATCTGT(+) |  | (Yang et al., 2017) |
|  | 2 | 16S | | CCACGATTACTAGCGATTCCGACTTC(-) |  |  |
|  | 1 | gltA | | ATGATCCGGGGTTCCTGTC (+) | 930 bp | (Guo et al., 2018) |
|  | 2 | gltA | | TGCAGGTCTGAGATAACCT (+) |  |  |
|  | 1, 2 | gltA | | TACAATACCGGAGTAAAAGT (-) |  |  |
|  | 1 | groEL | | TGAAGAGCATCAAACCCGAAG (+) | 874 bp | (Yang et al., 2017) |
|  | 1 | groEL | | CTRCTCGTGATGCTATCGG (-) |  |  |
| *A. platys* | 1 | 16S | | TCCTGGCTCAGAACGAACGCTGGCG(+) | 900 bp | (Barlough et al., 1996) |
|  | 1 | 16S | | AGTCACTGACCCAACCTTAAATGGCTG(-) |  |  |
|  | 2 | 16S | | GTCGAACGGATTATTCTTTATAGCTTGC(+) |  |  |
|  | 2 | 16S | | CCCTTCCGTTAAGAAGGATCTAATCTCC(-) |  |  |
|  | 1 | gltA | | TGRAAGAAAAWGCTGTTTTG(+) | 870 bp | (Guo et al., 2016) |
|  | 2 | gltA | | AGCTRTTTTRGAGTGYGGAG(+) |  |  |
|  | 1,2 | gltA | | GCTCTRGGRTCATARCTYTT (-) |  |  |
|  | 1 | groEL | | AGTCGATTAGGGAAGTAGTAC(+) | 1000 bp |  |
|  | 1 | groEL | | AGGATGGCTACAAGGTAATG(+) |  |  |
|  | 1,2 | groEL | | GCGTCCTCTACTCTGTCTT(-) |  |  |
| *A. marginale* | 1,2 | 16S | | GGATAGCCACTRGAARTGGTG(+) | 900 bp |  |
|  | 1 | 16S | | CGTGCTGACTTGACATCAT(-) |  |  |
|  | 2 | 16S | | CATCTCACGACACGAGCTG(-) |  |  |
|  | 1 | 16S | | CTGTCTGGTCCGGTACTGAC(+) | 700 bp |  |
|  | 2 | 16S | | TGGTCCGGTACTGACRCT(+) |  |  |
|  | 1,2 | 16S | | TGCCTCCTTDCGGTTGGC(-) |  |  |
|  | 1 | gltA | | TGGTAGAAAAAGCGATTTTAG(+) | 1200 bp |  |
|  | 2 | gltA | | ATAAGCTTGCCCGTTATGC(+) |  |  |
|  | 1,2 | gltA | | CCGGTATAAAGTTGGCGT(-) |  |  |
|  | 1 | groEL | | ACATGCTCCATACTGACTGC(+) | 860 bp |  |
|  | 2 | groEL | | AGATGAGATTGCACAGGTTG(+) |  |  |
|  | 1,2 | groEL | | AGATGCAAGCGTGTATAGCAG(-) |  |  |
| *A. bovis* | 1 | 16S | | TCCTGGCTCAGAACGAACGCTGGCG(+) | 900 bp | (Barlough et al., 1996) |
|  | 1 | 16S | | AGTCACTGACCCAACCTTAAATGGCTG(-) |  |  |
|  | 2 | 16S | | GTCGAACGATTATTCTTTATAGCTTGC(+) |  |  |
|  | 2 | 16S | | CCCTTCCGTTAAGAAGGATCTAATC(-) |  |  |
|  | 1 | gltA | | TTYATAGATGGRGATRAGGGC(+) | 1000 bp | (Guo et al., 2020) |
|  | 2 | gltA | | AGATGGRGATRAGGGCATYCT(+) |  |  |
|  | 1,2 | gltA | | AHCATTTCATRCCAYTGRG(-) |  |  |
|  | 1,2 | groEL | | ACTGCTGGACCGAAGGGCTT(+) | 850 bp |  |
|  | 1 | groEL | | CAAAGTGATGTCCTCCATCT(-) |  |  |
|  | 2 | groEL | | GCTATGTCRCCVAGCATGTCT(-) |  |  |
|  | 1 | groEL | | TTGCTAAATCTGGAAGRCCAC(+) | 800 bp |  |
|  | 2 | groEL | | GARGACGTTGAGGGTGAAGC(+) |  |  |
|  | 1,2 | groEL | | CATAAATACYGCCGCRAGAG(-) |  |  |
| *Ehrlichia* | 1,2 | 16S | | GAATAGCCATTAGAAATGATG(+) | 500 bp | (Guo et al., 2016) |
|  | 1 | 16S | | GTCAGTATCGAACCAGATAG(-) |  |  |
|  | 2 | 16S | | GTATCGAACCAGATAGCCG(-) |  |  |
|  | 1 | 16S | | CGGCTATCTGGTTCGATAC(+) | 750 bp |  |
|  | 2 | 16S | | CTATCTGGTTCGATACTGAC(+) |  |  |
|  | 1,2 | 16S | | GCTTCCTTKCGGTTAGCAC(-) |  |  |
|  | 1 | gltA | | CAGGHTTTATGTCWACTGCTGCT (+) | 1000 bp | (Loftis et al., 2004) |
|  | 2 | gltA | | TTATGTCWACTGCTGCTTGTGA (+) |  |  |
|  | 1,2 | gltA | | TAYAAYTGACGWGGACGACAT (-) |  |  |
|  | 1 | groEL | | TGGGCTGGYAATGAAATTGA(+) | 1100 bp | (Lu et al., 2022) |
|  | 2 | groEL | | AACATGGCAAATGTAGTTGT(+) |  |  |
|  | 1,2 | groEL | | TCAACAGCAGCTCTAGTTG(-) |  |  |
| TICK | 1 | COI | | GGTCAACAAATCATAAAGATATTGG(+) | 709 bp | (Cao et al., 2003) |
|  | 1 | COI | | TAAACTTCAGGGTGACCAAAAAATCA(-) |  |  |

**References**

Barlough, J.E., Madigan, J.E., Derock, E., and Bigornia, L. (1996). Nested polymerase chain reaction for detection of *Ehrlichia equi* genomic DNA in horses and ticks (*Ixodes pacificus*). *Vet Parasitol*. 63: 319-329. doi:10.1016/0304-4017(95)00904-3

Cao, W.C., Zhao, Q.M., Zhang, P.H., Yang, H., Wu, X.M., Wen, B.H., et al. (2003). Prevalence of *Anaplasma phagocytophila* and *Borrelia burgdorferi* in *Ixodes persulcatus* ticks from northeastern China. *Am J Trop Med Hyg*. 68: 547-550. doi:10.4269/ajtmh.2003.68.547

Guo, W.P., Huang, B., Zhao, Q., Xu, G., Liu, B., Wang, Y.H., et al. (2018). Human-pathogenic *Anaplasma* spp., and *Rickettsia* spp. in animals in Xi'an, China. *PLoS Negl Trop Dis*. 12: e0006916. doi:10.1371/journal.pntd.0006916

Guo, W.P., Tian, J.H., Lin, X.D., Ni, X.B., Chen, X.P., Liao, Y., et al. (2016). Extensive genetic diversity of Rickettsiales bacteria in multiple mosquito species. *Sci Rep*. 6: 38770. doi:10.1038/srep38770

Guo, W.P., Tie, W.F., Meng, S., Li, D., Wang, J.L., Du, L.Y., et al. (2020). Extensive genetic diversity of *Anaplasma bovis* in ruminants in Xi'an, China. *Ticks Tick Borne Dis*. 11: 101477. doi:10.1016/j.ttbdis.2020.101477

Jafar Bekloo, A., Ramzgouyan, M.R., Shirian, S., Faghihi, F., Bakhshi, H., Naseri, F., et al. (2018). Molecular Characterization and Phylogenetic Analysis of *Anaplasma* spp. and *Ehrlichia* spp. Isolated from Various Ticks in Southeastern and Northwestern Regions of Iran. *Vector Borne Zoonotic Dis*. 18: 252-257. doi:10.1089/vbz.2017.2219

Loftis, A.D., Ross, D.E., and Levin, M.L. (2004). Susceptibility of mice (*Mus musculus*) to repeated infestation with *Amblyomma americanum* (Acari: Ixodidae) ticks. *J Med Entomol*. 41: 1171-1174. doi:10.1603/0022-2585-41.6.1171

Lu, M., Meng, C., Gao, X., Sun, Y., Zhang, J., Tang, G., et al. (2022). Diversity of Rickettsiales in *Rhipicephalus microplus* Ticks Collected in Domestic Ruminants in Guizhou Province, China. *Pathogens*. 11. doi:10.3390/pathogens11101108

Yang, J., Liu, Z., Niu, Q., Liu, J., Han, R., Guan, G., et al. (2017). A novel zoonotic *Anaplasma* species is prevalent in small ruminants: potential public health implications. *Parasit Vectors*. 10: 264. doi:10.1186/s13071-017-2182-9

**Supplementary Table 2.** The species, numbers, and origins of ticks collected from Jinzhai County.

| Origin | Tick species | | | | | Total |
| --- | --- | --- | --- | --- | --- | --- |
|  | *H. longicornis* | *H. flava* | *H. hystricis* | *R. microplus* | *A. testudinarium* |  |
| Goat | 385 | 9 | 10 | 67 | 0 | 471 |
| Cattle | 80 | 6 | 0 | 150 | 0 | 236 |
| Dog | 4 | 0 | 3 | 0 | 2 | 9 |
| Grassland | 80 | 11 | 42 | 29 | 2 | 164 |
| Total | 549 | 26 | 55 | 246 | 4 | 880 |

# Supplementary Figures


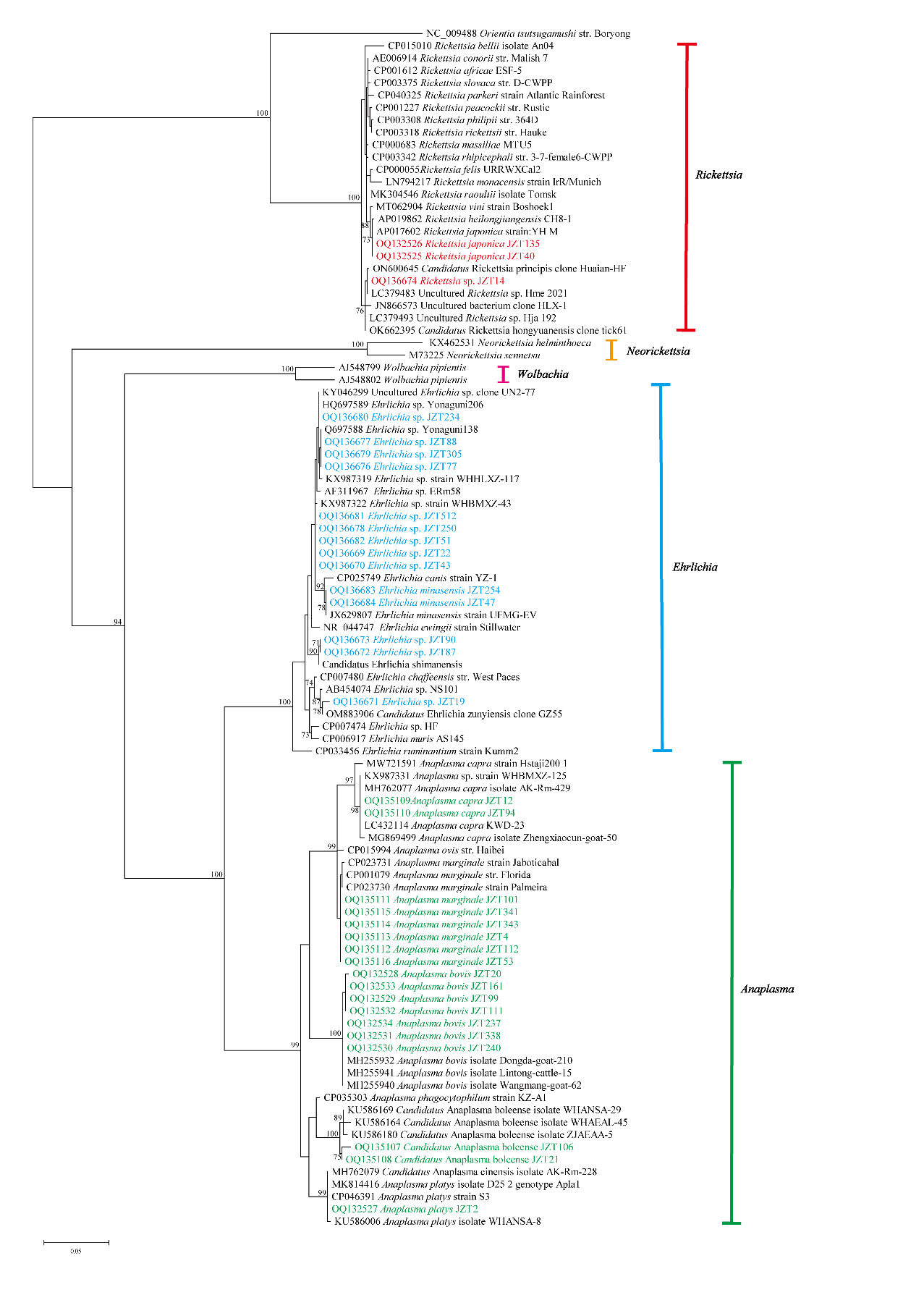


**Supplementary Figure 1.** Phylogenetic trees based on partial Rickettsiales 16S rRNA sequences using ML method with PhyML 3.0. Sequences of *Rickettsia* strains obtained in this study are marked in red; Sequences of *Ehrlichia* strains obtained in this study are marked in blue; Sequences of *Anaplasma* strains obtained in this study are marked in green.


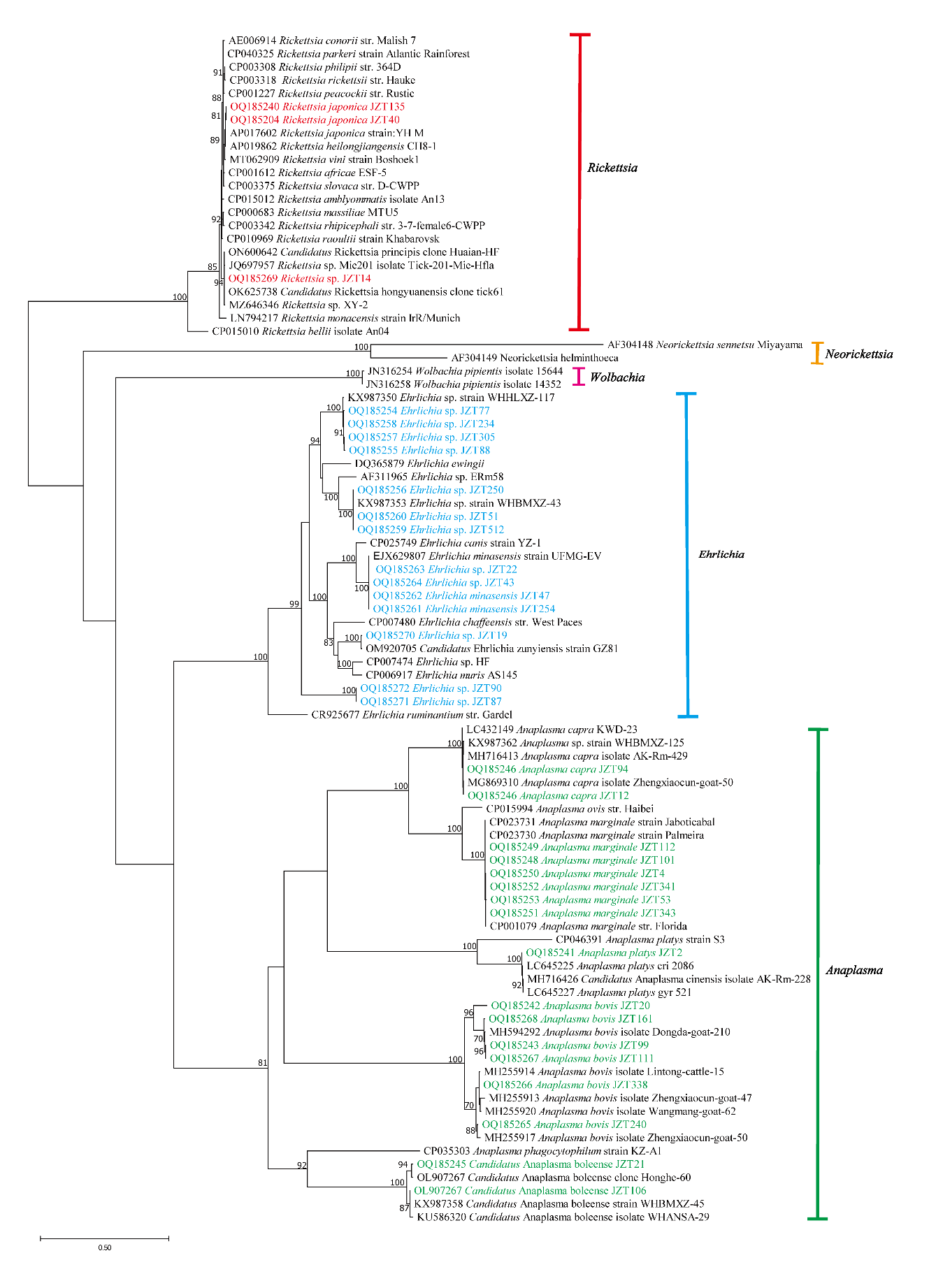


**Supplementary** **Figure 2.** Phylogenetic trees based on partial Rickettsiales *gltA* sequences using ML method with PhyML 3.0. Sequences of *Rickettsia* strains obtained in this study are marked in red; Sequences of *Ehrlichia* strains obtained in this study are marked in blue; Sequences of *Anaplasma* strains obtained in this study are marked in green.


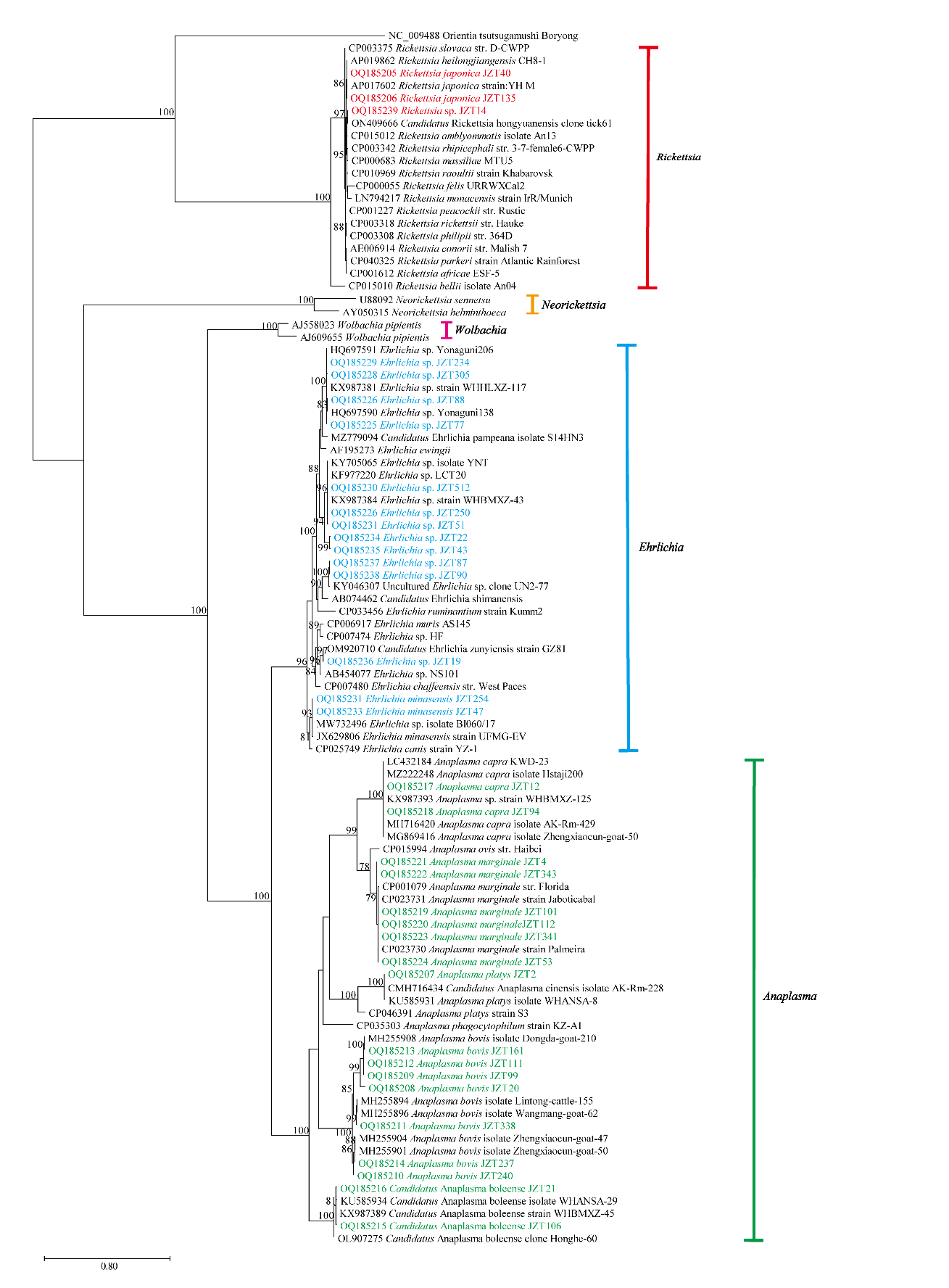


**Supplementary Figure 3.** Phylogenetic trees based on partial Rickettsiales *groEL* sequences using ML method with PhyML 3.0. Sequences of *Rickettsia* strains obtained in this study are marked in red; Sequences of *Ehrlichia* strains obtained in this study are marked in blue; Sequences of *Anaplasma* strains obtained in this study are marked in green.
